# The Critical Role of Logistics for Ageing-in-Place: Insights from Active Ageing Initiatives

**DOI:** 10.5334/ijic.10176

**Published:** 2026-01-28

**Authors:** Huay Ling Tay

**Affiliations:** 1 School of Business, Singapore University of Social Sciences, Singapore

**Keywords:** integrated care, ageing-in-place, operational integration, logistics, supply chain, Healthier SG

## Abstract

Singapore’s *Healthier SG* initiative represents a pivotal step toward integrated, preventive, and community-based care for an ageing population. While considerable attention has been given to integrating medical and social care, the role of logistics, ranging from service scheduling to last-mile delivery of health and mobility aids, remains under-addressed in policy implementation. Drawing on my experience as a logistics and health systems researcher, this paper posits that logistics and supply chain systems form a “third pillar” of care integration, particularly for ageing in place. Using Singapore’s transition to Healthier SG as a case, I reflect on implementation gaps, system design flaws, and promising innovations.

## Context and Aim

Singapore is one of the world’s most rapidly ageing societies. By 2030, nearly one in four residents will be aged 65 or older [10]. This demographic shift is accompanied by an increase in care complexity, multimorbidity, and a greater demand for community and home-based services. In response, Singapore has embraced *ageing-in-place* as a core principle across health and social policy, aiming to support older adults in remaining at home and in their communities for as long as possible [4].

In 2023, the Ministry of Health launched *Healthier SG*, a national strategy aimed at shifting the healthcare system away from episodic, hospital-centric care toward preventive, longitudinal, and community-based models [1]. Key pillars of this initiative presented in Table 1.

**Table 1:** Key Pillars of the Healthier SG Initiative.


PILLAR	DESCRIPTION

Enrolling residents with family doctors	Long-term primary care

Developing individualised health plans	Individualised health plans

Strengthening community partnerships	Active Ageing Centres (AACs) and regional networks

Promoting digital engagement	HealthHub and the Healthy 365 app


While the strategy emphasises medical-social integration, I argue that an equally important, yet less recognised, element is operational integration, particularly the role of logistics and supply chains in enabling continuity of care and self-management.

This paper is written from the perspective of a logistics and operations management researcher who has contributed to integrated care implementation projects, curriculum development, and national discussions on health system transformation. I reflect on Singapore’s experience in operationalising Healthier SG and the systemic importance of logistics in realising active ageing and ageing-in-place.

## Brief Description of Topic at Hand

Ageing-in-place requires more than clinical support. It demands reliable systems to ensure that medical supplies, meals, mobility aids, social activities, and even caregiver availability are delivered effectively to seniors at home. When logistical systems break down, when a wheelchair cannot be repaired, when transport to an AAC is unavailable, or when meals are not delivered, older adults become vulnerable to isolation, health deterioration, and unnecessary hospitalisation [7].

Despite these realities, logistics is often treated as a background function, divorced from care planning. Care coordinators, nurses, and social workers are often left to manually arrange logistics, navigating a complex web of vendors, community services, and informal caregivers [5].

The Healthier SG framework, while comprehensive in its medical and community care strategies, currently lacks an explicit design for logistics integration. This gap is increasingly apparent in regional pilot evaluations, community site observations, and conversations with care team members [8].

### Operational Integration Versus Functional Integration

A crucial distinction must be made between ‘operational integration’ and the more familiar ‘functional integration’ in care systems. Functional integration is typically understood as aligning support functions, such as ICT, finance, and HR across organisations, with a particular focus on interoperable digital systems [12]. However, the literature [5,8], notes that functional integration should also include logistics, such as service scheduling and inventory management. In practice, however, logistics often remains siloed, relegated to on-the-ground execution and excluded from strategic integration planning.

Operational integration, by contrast, is about the real-time coordination of resources, people, and processes to deliver services when and where needed [13]. It bridges the gap between digital coordination and on-the-ground delivery, ensuring, for example, that a nurse arrives promptly with the required equipment or that mobility aids reach clients without delay.

In this way, operational integration builds upon functional integration by making logistics a core part of care delivery, requiring attention in system design, workforce development, and policy. Without this, digital visibility alone cannot guarantee seamless, reliable care for older adults [14].

## Discussion and Reflection

### Logistics as the “Third Pillar” of Integration

Singapore’s integrated care framework has traditionally focused on linking medical and social services. However, as the care locus shifts to homes and neighbourhoods, I propose a third critical layer: **operational integration**—the embedding of logistics and supply chain coordination into the design and delivery of care.

AACs, for instance, coordinate diverse services such as exercise programs, medication reminders, and community screenings. However, these services often depend on unpredictable transport availability, insufficient manpower, or a lack of real-time information. AAC staff could spend considerable time managing transportation schedules, coordinating the delivery of walking aids, or verifying whether an older adult has received home-delivered meals or medications [3].

A well-integrated logistics system would enable proactive service routing, automated inventory tracking, and timely alerts in the event of disruptions.

### Singapore-Specific Illustrations and Lessons

The COVID-19 pandemic stress-tested Singapore’s care logistics. Programs such as the *Home Recovery Programme* (HRP) and *mask distribution campaigns* under *SupplyAlly* showcased the nation’s ability to mobilise and deliver essential services at scale, with remarkable speed and coordination. These temporary systems revealed what is possible when logistics is treated as a first-order system design concern [2,6]. These efforts revealed what is possible when logistics is treated as a core system component, enabling decentralised care without compromising safety. However, scaling such logistics capabilities outside a crisis context remains challenging.

Outside the pandemic, Singapore’s regional health systems continue to face operational challenges. In the pilots I observed, community nurses from transitional care teams had to cancel or delay appointments due to the unavailability of patient transport. Other cases involved significant delays in delivering mobility aids and care items prescribed under integrated care plans, due to gaps in supplier coordination and a lack of effective tracking systems.

These are not clinical failures; they are **logistical failures**. As Healthier SG seeks to extend care from hospitals into the community, these examples underscore the importance of treating logistics as a foundational enabler of continuity and equity in care delivery.

### Reframing Supply Chain Management in the Context of Ageing

Singapore’s strengths in port logistics, e-commerce, and just-in-time supply chains provide a robust foundation for building a parallel model for health and ageing services. Several supply chain principles are directly applicable as shown in Table 2.

**Table 2:** Supply Chain Principles for Ageing-in-Place and Community Care.


ASPECT	DESCRIPTION

Last-mile fulfilment	Ageing-in-place depends on ensuring the timely delivery of home-based services [5]

Demand forecasting	Community-level needs can be modelled using predictive analytics, improving readiness and reducing supply lags

Orchestration and coordination	Aligning rehabilitation, transport, equipment delivery, and wellness visits into coordinated bundles

Inventory and service visibility	Just as e-commerce provides real-time tracking, ageing services should allow stakeholders to track logistics fulfilment


GovTech’s *SupplyAlly* platform, originally deployed for mask distribution, demonstrates the potential of digital logistics tracking in health delivery [6]. Similar platforms could integrate with *HealthHub* to allow care teams and residents to view both health status and logistics readiness.

### Embedding Logistics into Workforce and Governance Structures

Operational integration requires new workforce capabilities. Today, care coordinators juggle social assessments, clinical triage, and logistics management, often without dedicated tools or training. This is unsustainable.

New roles, such as **care logistics coordinators** or **community operations planners** can be introduced. These professionals can handle routing, supplier scheduling, and inventory assurance, freeing clinical and social professionals to focus on person-centred care. Training programs at institutions can develop foundational logistics competencies for the community care sector [9].

Recent research has also demonstrated how Lean Management and Dynamic Capabilities Theory can be applied in the voluntary and community sector (VCS) to enhance agility and optimise resources in integrated care delivery. These frameworks offer practical tools for community partners to manage limited logistics capacity, eliminate bottlenecks, and adapt to unpredictable service demands—particularly crucial for last-mile care in low-resource neighbourhoods [11].

Additionally, logistics must be included in regional care planning and budgeting. Regional Health Groups (RHGs) can map care logistics capacity across partners, track fulfilment metrics, and establish contingency plans for service disruptions [7].

### Community-Enabled Logistics: Scaling Informal Networks

During the pandemic, community networks comprising grassroots leaders, mutual aid groups, and volunteers filled vital logistical gaps. These networks can be sustained and expanded under Healthier SG.

For instance, logistics volunteers at Active Ageing Centres can assist with meal drops, escort services, or light repair tasks. Mobile logistics nodes such as community-based storage hubs for equipment and supplies can be piloted in mature estates with high ageing density. Community Silver Trust funding can support these innovations [2].

**Figure 1:**
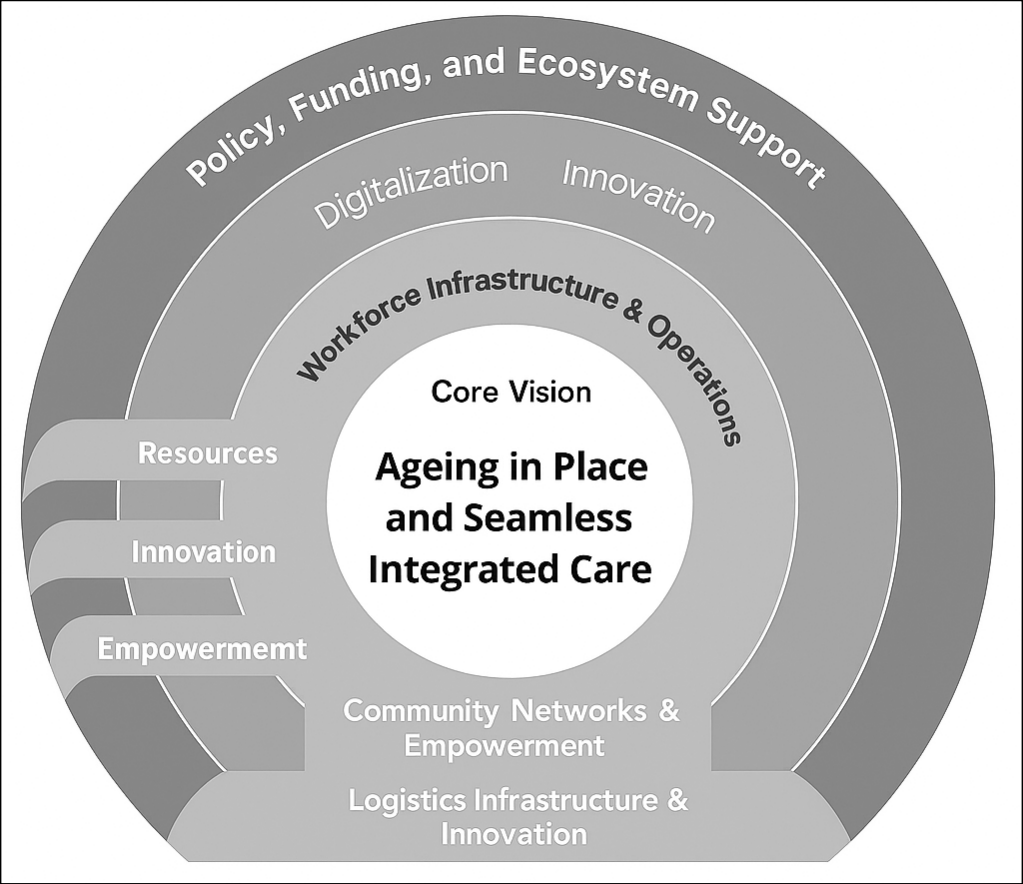
The Community Care Logistics Integration Model.

The **Community Care Logistics Integration Model** (Figure 1) is a conceptual framework that encapsulates the key domains required to support ageing-in-place and integrated care in Singapore. Structured as four concentric layers surrounding a central vision, the model emphasises the systemic integration of logistics, community capacity, workforce development, and policy support.

At the heart of the Community Care Logistics Integration Model is the vision of “Ageing in Place and Seamless Integrated Care.” The first layer, **Community Networks & Empowerment**, highlights the significance of grassroots actors, volunteers, and mutual aid groups that bridge critical last-mile service gaps through initiatives such as meal delivery, transportation assistance, and home support. The next layer, **Logistics Infrastructure & Innovation**, highlights the role of mobile hubs, digital platforms like SupplyAlly, and logistics strategies that encompass demand forecasting and last-mile fulfilment to ensure timely and efficient care delivery.

**Workforce & Operations** comprises the third layer, emphasising new roles such as care logistics coordinators, bolstered by targeted training in logistics and Lean operations to integrate clinical, social, and logistical service needs. Finally, **Policy, Funding & Ecosystem Support** forms the outermost layer, advocating for logistics to be prioritised in policy, the utilisation of innovation ecosystems, and enabling Regional Health Groups (RHGs) to coordinate resource allocation and contingency plans. Throughout the model, cross-cutting themes of digitalisation, innovation, and empowerment thread through every layer, illustrating the dynamic interconnections required to create a resilient, truly **integrated community care logistics system**.

### International Relevance and Lessons for Other Countries

Singapore’s experience offers important insights for health and care systems worldwide grappling with ageing populations and the shift toward community-based care. Despite advancements in functional integration and ICT adoption, logistics remains frequently overlooked [14,15].

Embedding logistics as a strategic design principle in integrated care policy, as seen in Singapore’s pandemic response, enables rapid scaling and innovation when system coordination is prioritised [2]. Investing in logistics workforce development and digital platforms that facilitate both clinical and logistical integration is essential; countries like Japan, the Netherlands, and Germany, with robust logistics sectors, can adapt these principles effectively [16].

Empowering communities to co-deliver logistics services, especially in neighbourhoods with high ageing density, fosters resilience. Singapore’s mobilisation of volunteers for last-mile fulfilment underlines the impact of community networks [17].

Ultimately, recognising logistics as a foundational enabler, not simply an operational support, can help health systems realise the promise of ageing in place and integrated care.

## Conclusion and Recommendations

Singapore’s Healthier SG initiative marks a bold reconfiguration of healthcare delivery, grounded in prevention, personalisation, and community engagement. However, the capacity to operationalise ageing-in-place depends on a largely invisible infrastructure: **logistics and supply chain systems**.

My reflections suggest five key actions for Singapore and other systems pursuing integrated care:

- **Elevate logistics to a core design principle** in integrated care policy and Regional Health Groups (RHGs) planning.
- **Invest in workforce development** for logistics coordination roles across the community and health teams.
- **Digitally integrate supply, scheduling, and fulfilment systems** with clinical and social care records.
- **Leverage Singapore’s logistics innovation ecosystem** (e.g., GovTech, logistics SMEs) to co-develop platforms for community health logistics.
- **Empower local communities to co-deliver logistics services**, especially in mature and rental estates with high proportions of older residents.

Logistics may be invisible, but its failure is not. By integrating supply chains into the heart of integrated care, Singapore can truly realise the promise of ageing in place, not only as a policy aspiration, but as a lived, supported experience for all seniors.
